# Azathioprine induction of tumors with microsatellite instability: Risk evaluation using a mouse model

**DOI:** 10.18632/oncotarget.4638

**Published:** 2015-08-17

**Authors:** Sahra Bodo, Magali Svrcek, Isabelle Sourrouille, Peggy Cuillières-Dartigues, Tatiana Ledent, Sylvie Dumont, Laetitia Dinard, Philippe Lafitte, Camille Capel, Ada Collura, Olivier Buhard, Kristell Wanherdrick, Alexandra Chalastanis, Virginie Penard-Lacronique, Bettina Fabiani, Jean-François Fléjou, Nicole Brousse, Laurent Beaugerie, Alex Duval, Martine Muleris

**Affiliations:** ^1^ INSERM, UMR_S 938, CDR Saint-Antoine, Equipe « Instabilité des Microsatellites et Cancer », Equipe labellisée par la Ligue Nationale contre le Cancer, F-75012, Paris, France; ^2^ Sorbonne Universités, UPMC Univ Paris 06, UMR_S 938, CDR Saint-Antoine, F-75012 Paris, France; ^3^ AP-HP, Hôpital Saint-Antoine, Service d'Anatomie Pathologique, F-75012 Paris, France; ^4^ AP-HP, Institut Gustave Roussy, Service d'Anatomie Pathologique, F-94805 Villejuif, France; ^5^ IFR 65, F-75012 Paris, France; ^6^ INSERM, U985, Institut Gustave Roussy, F-94805 Villejuif, France; ^7^ AP-HP, Hôpital Necker-Enfants Malades, Service d'Anatomie Pathologique, F-75015 Paris, France; ^8^ AP-HP, Hôpital Saint-Antoine, Service de Gastroentérologie, F-75012 Paris, France

**Keywords:** pharmacogenetics, iatrogenic cancer, microsatellite instability, thiopurine tolerance, azathioprine

## Abstract

Mismatch-repair (MMR)-deficient cells show increased *in vitro* tolerance to thiopurines because they escape apoptosis resulting from MMR-dependent signaling of drug-induced DNA damage. Prolonged treatment with immunosuppressants including azathioprine (Aza), a thiopurine prodrug, has been suggested as a risk factor for the development of late onset leukemias/lymphomas displaying a microsatellite instability (MSI) phenotype, the hallmark of a defective MMR system. We performed a dose effect study in mice to investigate the development of MSI lymphomas associated with long term Aza treatment. Over two years, Aza was administered to mice that were wild type, null or heterozygous for the MMR gene *Msh2*. Ciclosporin A, an immunosuppressant with an MMR-independent signaling, was also administered to *Msh2^wt^* mice as controls. Survival, lymphoma incidence and MSI tumor phenotype were investigated. *Msh2^+/−^* mice were found more tolerant than *Msh2^wt^* mice to the cytotoxicity of Aza. In *Msh2^+/−^* mice, Aza induced a high incidence of MSI lymphomas in a dose-dependent manner. In *Msh2^wt^* mice, a substantial lifespan was only observed at the lowest Aza dose. It was associated with the development of lymphomas, one of which displayed the MSI phenotype, unlike the CsA-induced lymphomas. Our findings define Aza as a risk factor for an MSI-driven lymphomagenesis process.

## INTRODUCTION

Substantial clinical progress in immunosuppression and anticancer chemotherapy has improved the overall survival of patients. Unfortunately, the survival benefits conferred by some long-term treatments such as thiopurine are also associated with an increased risk of developing therapy-related cancers. Thiopurine drugs comprise azathioprine (Aza) and its metabolite 6-mercaptopurine. Aza is widely used as an immunosuppressive agent for the prevention of transplant graft rejection and for autoimmune diseases such as inflammatory bowel disease (IBD) and autoimmune hepatitis. 6-mercaptopurine is commonly used as part of maintenance therapy in childhood acute lymphoblastic leukemia. Aza is classified as a human carcinogen but the specific mechanisms that underlie its *in vivo* carcinogenicity following thiopurine therapy remain unclear.

Interestingly, several clinical studies have reported an association between the use of prolonged thiopurine regimens and the occurrence of tumors displaying microsatellite instability (MSI). The MSI tumor phenotype is the hallmark of a defective DNA mismatch repair (MMR) system, the major function of which is to edit errors produced during replication. Inactivation of the MMR system greatly increases spontaneous mutation rates. This is observed as MSI and manifests as frequent deletion mutations in DNA regions comprising mostly of long mono- or dinucleotide tracts. *In vitro* studies have demonstrated that MMR also plays an important role in thiopurine-mediated cytotoxicity, as evidenced by the increased tolerance of MMR-deficient cells to thiopurine exposure. The major cytotoxic effects of thiopurine drugs occur following incorporation of thio-guanine (TG) into DNA and subsequent misincorporation of thymidine opposite TG during DNA replication. The aberrant processing of these DNA base pairs by MMR generates potentially lethal DNA lesions. MMR–deficient cells are unable to initiate lethal processing of the TG-containing base pairs and escape cytotoxicity. Owing to this resistance conferred by defective MMR, known as thiopurine tolerance, it has been suggested that thiopurines may constitute a risk factor for development of human cancer via selection of MMR-defective cells that subsequently undergo neoplastic cell transformation following the accumulation of genetic mutations caused by MSI (for review [1]).

Despite *in vitro* functional data suggesting a putative causal role for thiopurine therapy in the development of MSI cancers, the clinical evidence to date is mixed and hence this remains to be established conclusively. An MSI phenotype was observed in acute myeloid leukemia/myelodysplastic syndromes that developed after organ transplantation and were associated with immunosuppression by Aza therapy [2]. Previous studies from our group [3, 4] also reported a low frequency of the MSI phenotype in immunodeficiency-related non-Hodgkin lymphomas arising in transplant patients who received Aza as part of their immunosuppressive regimen (13/143 *vs* 0/500 in immunocompetent patients). However, some patients with MSI tumors of suspected iatrogenic origin did not receive thiopurine therapy [4]. Interpretation of these findings is further complicated by the fact that immunosuppressive regimens in transplant patients often combine Aza with other drugs such as steroids and ciclosporin A (CsA). Additionally, the MSI phenotype was not detected in immunodeficiency-related skin cancers and no significant association was observed between Aza intake and MSI in intestinal neoplasias arising in patients with IBD [5–8]. Thus, the clinical association between thiopurine treatment-related cancer and MMR defects remains uncertain and requires further investigation.

Here we describe results using an animal model to address whether thiopurine tolerance is the putative oncogenic mechanism that underlies the emergence of MSI tumors. In an earlier proof of concept study we demonstrated that Aza induced massive and early development of MSI lymphomas in *Msh2^+/–^* mice carrying a germline mutation in the MMR gene *Msh2* [9]. In contrast, untreated *Msh2^+/–^* mice developed MSI lymphomas at a very low frequency. This work established the *Msh2* mouse model, the murine equivalent of Lynch syndrome in humans, as relevant for the investigation of thiopurine tolerance as a causal mechanism for MSI cancers. However, the substantial toxicity of Aza in *Msh2* wild type (*Msh2^wt^*) mice prevented us from investigating the ability of this agent to induce MSI-driven malignancy in mice without a genetic predisposition to MMR defects. Moreover, we did not investigate other immunosuppressants whose action is independent of MMR. In the present work we have therefore performed a dose effect study using wild type, null or heterozygous mice for the *Msh2* gene and identified appropriate long-term treatment conditions for Aza, thus avoiding the major cytotoxic effects encountered previously. We hypothesized that an Aza dosage that enables a substantial survival would allow the development of lymphomas, a small proportion of which would be of MSI phenotype, as observed in humans. To evaluate whether the observed effects could be attributed specifically to Aza, we also investigated CsA, another frequently prescribed immunosuppressant that inhibits the transcription of cytokines, leading to reduced functional ability of effector T-cells.

## RESULTS

### Impact of azathioprine on *Msh2*^+/–^ and *Msh2^ko^* mice

**Figure 1:**
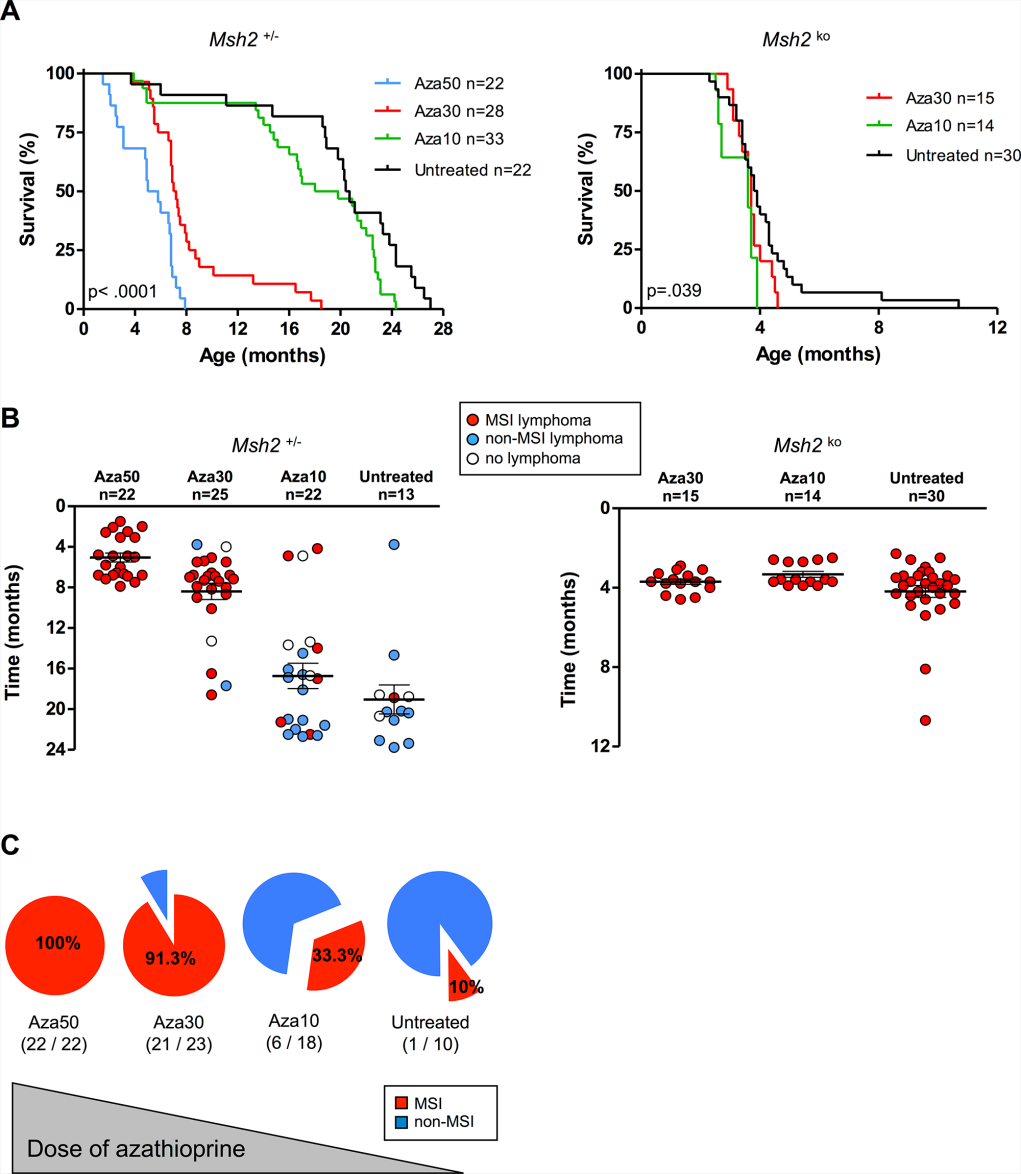
Effect of azathioprine treatment on survival, lymphoma incidence and MSI tumor phenotype in Msh2^*+/−*^ and Msh2^*ko*^ mice **A.** Evidence for a dose-response relationship between azathioprine treatment and the survival of *Msh2^+/–^* mice: Kaplan–Meier survival analysis of untreated and azathioprine-treated *Msh2^+/–^* mice (left) and *Msh2^ko^* mice (right). Survival curves for *Msh2^+/–^* mice after Aza50 treatment are from our previous study [9]. All *P* values are two-sided. Log-rank test. **B.** Lymphoma occurrence and delay of onset in the different mice cohorts according to *Msh2^+/–^* (left) or *Msh2^ko^* (right) genotype. Mean age of lymphoma onset and standard error of the mean are indicated. **C.** Evidence for a dose-response relationship between azathioprine treatment and incidence of MSI phenotype in lymphomas of *Msh2^+/–^* mice. Aza10 = azathioprine 10 mg/L; Aza30 = azathioprine 30 mg/L; Aza50 = azathioprine 50 mg/L.

We first specified the way Aza treatment could trigger MSI lymphomagenesis in the context of a genetic predisposition to MSI tumor development (i.e. *Msh2^+/–^*). A dose-response relationship was observed for *Msh2^+/–^* mice, with median survival of 164, 220 and 575 days for Aza 50, 30 and 10 mg/L treatment, respectively (*P* < .0001; Log-rank test) (Figure 1A). Aza50-treated *Msh2^+/–^* mice systematically developed early onset, MSI lymphomas (22/22 *vs* 1/10 for untreated *Msh2^+/–^* animals) (Figure 1B). The onset of tumors was significantly delayed at lower Aza doses, with a mean age of onset of 154, 255 and 539 days for Aza50, Aza30 and Aza10, respectively (*P* < .0001; ANOVA test). The incidence of the MSI phenotype was also significantly reduced with the lowest Aza dose (21/23 and 6/18 for Aza30 and Aza10, respectively *P* < .0001; Fisher's exact test) (Figure 1C). Compared to untreated *Msh2^+/–^* mice, Aza10-treated mice showed a slightly shorter median survival (575 *vs* 625 days, respectively; *P* = .020; Log-rank test) but comparable median age of lymphoma onset (539 *vs* 576 days, respectively; *P* = .59; Student's t test).

As expected, all control *Msh2^ko^* mice, whether treated or not, were sacrificed before the age of 11 months. The median survival of *Msh2^ko^* mice was 117 days for untreated animals, 113 days with Aza30 and 109 days with Aza10 (*P* = .039; Log-rank test) (Figure 1A). As expected, all *Msh2^ko^* mice developed patent tumor syndromes, *i.e*. abnormal enlargement of the thymus, spleen or liver and massive and diffuse proliferation of lymphoma cells, that displayed MSI (Figure 1B).

### Impact of azathioprine and ciclosporin A on *Msh2^wt^* mice

**Figure 2:**
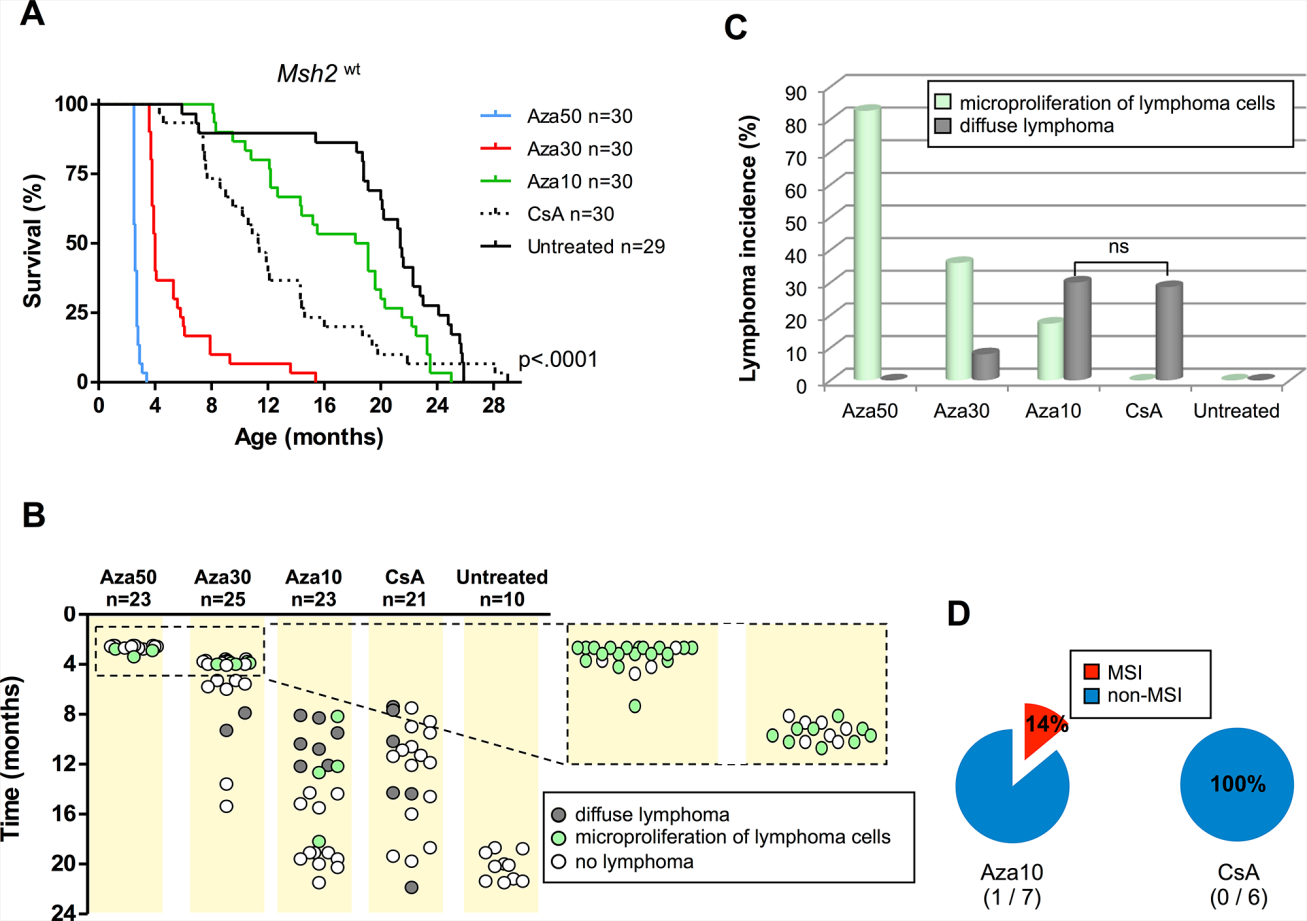
Effect of azathioprine and ciclosporin A treatments on survival, lymphoma incidence and MSI tumor phenotype in Msh2^*wt*^ mice **A.** Evidence for a dose-response relationship between immunosuppressive treatment and the survival of *Msh2^wt^* mice: Kaplan–Meier survival analysis of untreated, azathioprine- and ciclosporin A-treated *Msh2^wt^* mice. All *P* values are two-sided. Log-rank test. **B.** Occurrence of lymphoproliferations and delay of onset in the different cohorts of *Msh2^wt^* mice. **C.** Lymphoma incidence. Fisher's exact test; ns=not significant. **D.** Incidence of MSI phenotype in lymphomas. Aza10 = azathioprine 10 mg/L; Aza30 = azathioprine 30 mg/L; Aza50 = azathioprine 50 mg/L; CsA = ciclosporin A 300 mg/L.

Similar to *Msh2^+/–^* mice, a dose-response relationship was observed between Aza treatment and the survival of *Msh2^wt^* mice. The lowest Aza dose was associated with a significantly longer lifespan, with median survival of 78,123 and 568 days for Aza50, Aza30, and Aza10, respectively (*P* < .0001; Log-rank test) (Figure 2A). Whereas Aza10 was the only dose compatible with a substantial survival of animals, it still demonstrated a significant impact upon lifespan (568 *vs* 652 days for untreated mice; *P* = .007; Log-rank test). A patent tumor syndrome was observed in a substantial proportion of the Aza10 cohort (7/23, 30.5%) (Figures 2B and 2C). *Msh2^wt^* mice exposed to higher Aza doses (Aza30 and Aza50) were sacrificed within several months of birth due to poor tolerance to therapy. Consistent with our previous results [9], micro-lymphoproliferations were frequently observed during histological examination of these animals (Figures 2B, 2C). Microscopic foci comprised of medium-to-large neoplastic lymphoid cells were restricted to the spleen area located under the capsule.

CsA-treated *Msh2^wt^* mice also showed significantly shorter survival compared to untreated mice (345 *vs* 652 days, respectively; *P* = .002; Log-rank test) (Figure 2A). Aza10- and CsA-treated *Msh2^wt^* mice showed comparable survival (median of 568 *vs* 345 days, respectively; *P* = .045; Log-rank test) and both groups showed the presence of lymphomas in about one third of cases (7/23 *vs* 6/21, respectively; *P* = .9; Chi2 test) (Figures 2B and 2C).

### MSI status of azathioprine- and ciclosporin A-induced lymphomas in *Msh2^wt^* mice

Microsatellite genotyping was performed in all *Msh2^wt^* mice that developed patent tumor syndrome. As described above, these had mostly been treated with Aza10 or CsA. A diffuse MMR-deficient lymphoma due to proliferation of MSI tumor cells was found in 1/7 Aza10-treated *Msh2^wt^* mice. At contrast, all tumor DNAs from the 6 CsA-treated mice with lymphoma were non-MSI (Figure 2D). Two Aza30-treated mice that had not been sacrificed at a young age, showed a late onset lymphoma syndrome that was also non-MSI. Tumor cells obtained by laser microdissection from splenic microproliferations in four Aza50-treated *Msh2^wt^* mice also failed to show MSI. Results of microsatellite instability analysis are detailed in the Supplementary Table S1.

**Figure 3:**
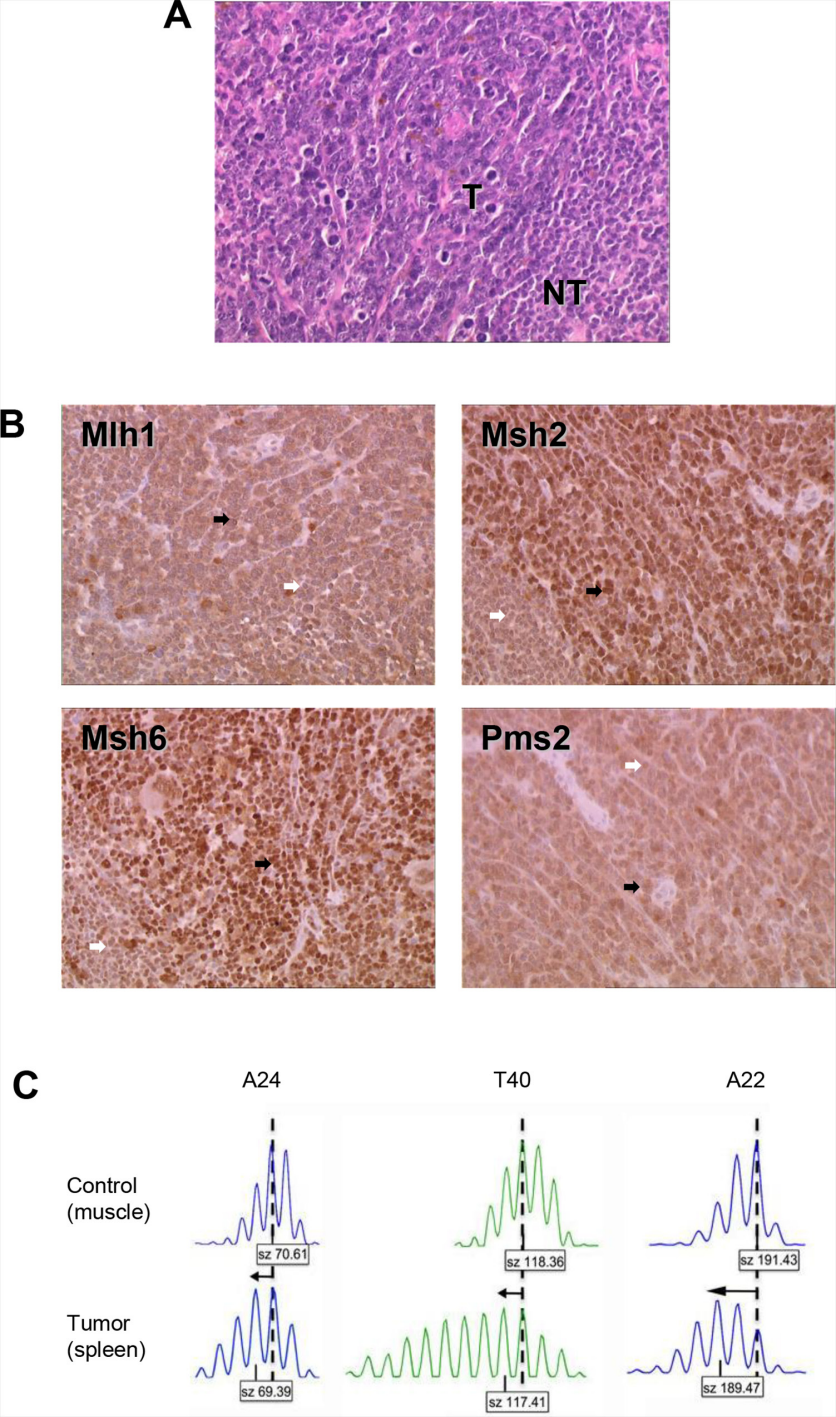
Characterization of the MSI lymphoma syndrome arising in Aza10-treated Msh2^*wt*^ mouse **A.** Histological characterization showing hematoxylin–eosin staining of splenic lymphoma. T indicates tumor cells; NT indicates normal lymphocytes. **B.** Immunohistochemistry of spleen tissue sections. Tumor cells (black arrows) stained positively. Normal lymphocytes (white arrows) showed less pronounced staining of MMR proteins due to their lower proliferative rate. Antibodies against Mlh1 and Pms2 provided less intense IHC staining however positive staining was retained in tumor cells (black arrows). An IHC picture of a non-MSI / MMR-proficient splenic lymphoma can be seen in the Supplementary Figure S2 to illustrate that technical point. **C.** Allelic profiles of the three noncoding microsatellite markers (A24, T40 and A22) displaying shorter alleles (arrows) for the three markers in tumor (spleen) compared to control (muscle) tissue. Dashed vertical lines indicate the predominant allele size observed in control DNA. Predominant allele size (in base pair) is indicated in the box below each profile.

The single *Msh2^wt^* mouse that developed an MSI tumor syndrome was sacrificed at the age of 315 days because of posterior leg paralysis. At autopsy, it presented an enlarged spleen and lymph nodes. Histological examination revealed massive (>90%) infiltration of the spleen, thymus and lymph node tissues by medium to large size lymphoid cells. Examination of tumor DNA from the splenic tissue with three microsatellite markers revealed a change in length of all three loci compared with normal tissue DNA from the same mouse (Figures 3A and 3C).

### Molecular mechanism underlying MMR-deficiency due to thiopurine tolerance in tumor cells

PCR-based genotyping was performed on all MSI tumors that developed in *Msh2*^+/−^ mice following an immunosuppressive regimen. In the majority of cases (22/26, including 19/21 Aza30- and 3/5 Aza10-treated *Msh2*^+/–^ mice), inactivation of the MMR system resulted from somatic loss of the remaining wild type *Msh2* allele (i.e. loss of heterozygosity), as previously reported [9]. cDNA sequencing of *Msh2* was performed in the tumor from the single *Msh2*^wt^ mouse that displayed an MSI lymphoma following Aza treatment, however no somatic mutation was detected. Immunohistochemical analysis of Msh2, Mlh1, Msh6 and Pms2 expression was then performed to further investigate the mechanism underlying loss of MMR function in the tumor from this *Msh2*^wt^ mouse. As shown in Figure 3B, the tumor cells stained positively for these four major MMR proteins.

## DISCUSSION

In the present study we investigated the ability of Aza, an immunosuppressant from the thiopurine family, to trigger MSI lymphomagenesis in mice. Aza induces apoptosis of MMR-proficient cells via the MMR–dependent signaling of Aza-induced DNA damage. Aza also has immunosuppressive properties due to induction of apoptosis in T cells [10] and inhibition of *de novo* purine biosynthesis which hampers cell division especially in lymphocytes, which unlike other cell types cannot use the salvage pathway of purine synthesis [11]. Importantly, the two latter effects are not dependent on MMR status. We previously observed considerably more toxicity at the higher dose of Aza (50 mg/L) in *Msh2^wt^* mice compared to *Msh2^+/−^* mice [9] and confirmed here that heterozygous mice are more tolerant to the cytotoxic effects of Aza (Supplementary Figure S1). These observations are consistent with an earlier report using another MMR murine model (*Mlh1* mice) that showed *Mlh1^+/–^* mice were less sensitive to the lethal effects of a methylating agent than *Mlh1^wt^* control mice (thiopurines and methylating agents share a common MMR processing mechanism for converting damaged DNA bases into lethal lesion) [12]. Our results also concur with *in vitro* experiments showing that immortalized lymphoblasts from Lynch patients who are heterozygous at the *MSH2* locus were more tolerant to the methylating agent temozolomide than *MSH2^wt^* cells [13].

Together, these observations suggest the overall clinical impact of Aza results from a combination of factors such as *Msh2* genotype, drug dosage and the balance between the cytotoxic and oncogenic properties of this drug. To account for the observed survival and tumor phenotypes in drug-treated mice with different *Msh2* genotype status, we propose the following explanations. First, all *Msh2^ko^* mice (untreated and Aza-treated) exhibited the characteristic development of MSI diffuse lymphomas that ultimately resulted in their early death. Whereas untreated *Msh2^+/–^* mice only rarely develop spontaneous MSI neoplasms, Aza induced a high incidence of MSI lymphomas in a dose-dependent manner in these animals. This is likely to be related to the burden of induced thiopurine DNA lesions and the resulting selective pressure to escape cell apoptosis through MMR inactivation. In most *Msh2^+/–^* mice, this inactivation occurs via somatic loss of the remaining wild type *Msh2* allele. In Aza-treated *Msh2^wt^* mice, a balance was observed between the cytotoxic and oncogenic properties of the drug. Survival and incidence of diffuse lymphomas were inversely proportional to the dose of azathioprine. We hypothesize that the microproliferations of lymphoma cells, which were mostly observed in Aza50- and Aza30-treated *Msh2^wt^* mice, did not lead to lymphoma because either their progression has been efficiently prevented by the immunosuppressive effect of Aza treatment or the mice died prematurely from organ failure related to the high drug cytotoxicity at this dosage. Aza10 and CsA treatments resulted in similar survival times and induction of lymphomas in a substantial and comparable proportion of *Msh2^wt^* mice. This mirrors the situation in humans where the development of lymphomas is a known adverse effect of immunosuppressive treatment, although the incidence of iatrogenic lymphomas is much higher in mice. An MSI tumor phenotype was observed in one *Msh2^wt^* mouse following Aza10 treatment. At contrast, none was observed in the CsA treated *Msh2^wt^* mice and we found that CsA treatment did not result in a significant induction of MSI lymphomas in *Msh2^+/–^* mice (data not shown). All together, our data support a causal role for Aza treatment, but not CsA treatment, in triggering MSI-driven lymphomagenesis in *Msh2^wt^* or *Msh2^+/–^* mice.

Considering that the risk of lymphoproliferative disorder (LPD) was five times higher in IBD patients treated with thiopurines than in those never exposed to these drugs [14], we searched for MSI phenotype in a series of 13 IBD-related LPD and found one case, which interestingly developed after thiopurine (imurel) therapy (Supplementary Table S2). Thus, this observation broadens the clinical contexts of thiopurine-associated lymphoproliferative disorders with MSI phenotype. Interestingly, as with the lymphoma that developed in the Aza10-treated *Msh2^wt^* mouse, the tumor cells from this MSI case were shown by immunohistochemistry to retain expression of the 4 MMR proteins (Supplementary Figure S3). This was also the case in about one third (3/8) of the immunodeficiency-related non-Hodgkin lymphomas with a MSI phenotype that develop in transplant patients treated with Aza, that we previously reported [4]. These findings suggest that new mechanisms for inactivation of MMR, which remain to be determined, are associated with clinical contexts of thiopurine treatment.

In summary, our study allows an estimation of the clinical risk associated with prolonged Aza intake based upon the development of iatrogenic tumors with MSI in a mouse model. Our data clearly demonstrate the ability of Aza to trigger lymphomagenesis through an MSI-driven process *in vivo* in the absence of genetic predisposition to MMR defects. The limitation of this study is the small number of *Msh2^wt^* mice that developed iatrogenic lymphoma with MSI but increasing the number of treated *Msh2^wt^* mice was not feasible from an ethical point of view. However, based upon post-transplant lymphoproliferative disorders in humans, such event was expected to be rare (9%) [3, 4]. Together, our findings could have important clinical implications for patients receiving azathioprine therapy and more broadly thiopurines. Therefore, we recommend systematic screening for MSI in lymphomas and other neoplasms that arise in patients treated with thiopurine drugs. Such screening could be particularly important because tumors with MSI usually have a different prognosis and sensitivity to chemotherapeutic agents compared with tumors that lack MSI, as has been shown for colorectal carcinomas [15].

## MATERIALS AND METHODS

### Mice and treatments

*Msh2^+/–^* mice on an FVB background [16] were intercrossed to obtain animals that were null, heterozygous, or wild type for the *Msh2* gene. Mice genotypes were determined by PCR analysis [17]. Aza was given orally *via* the drinking water using Imurel 50 mg (GlaxoSmithKline, Marly-le-Roi, France) to prepare a 50 mg/L, 30 mg/L or 10 mg/L drinking water solution (referred to as Aza50, Aza30 and Aza10 treatment in the text). The dose of azathioprine administered through the Aza50 treatment was estimated at 6–20 mg/kg body weight per day which corresponds to a human dose equivalent of 0.5–1.6 mg/kg body weight per day [18] and lies within the therapeutic range [19, 20]. Groups of mice received Aza10 (14 *Msh2^ko^*, 33 *Msh2^+/–^* and 30 *Msh2^wt^*) or Aza30 (15 *Msh2^ko^*, 28 *Msh2^+/–^* and 30 *Msh2^wt^*) and a group of 30 *Msh2^wt^* mice also received Aza50. Control mice (30 *Msh2^ko^*, 22 *Msh2^+/–^* and 29 *Msh2^wt^* mice) received only water to drink. A final group of 30 *Msh2^wt^* mice received drinking water that contained 300 mg/L ciclosporin A (CsA) (Neoral 100 mg/mL, Novartis Pharma SAS, Rueil-Malmaison, France). The dose of CsA administered to mice was estimated at 40–120 mg/kg body weight per day *i.e*. a human dose equivalent of 6.48–9.72 mg/kg body weight per day, which lies in the upper limit of the maintenance treatment administered to human transplant patients (*i.e*. 2 to 6 mg/kg according to Novartis recommendations). Food and drinking were *ad libitum*. All treatments were administered from the age of 6 weeks. Mice were kept in a pathogen-free environment and health was monitored daily. Animals that displayed signs of poor health, including breath insufficiency and posterior leg paralysis were sacrificed by cervical dislocation. The spleen, liver, thymus, lymph nodes and gut were removed for examination together with muscle (as source of germinal DNA). Since *Msh2*^wt^ mice on FVB background are prone to develop lymphomas of late occurrence, analysis was not continued beyond 24 months. All animal experiments were conducted in accordance with the regulations controlling procedures in live animals (approval Ce5/2010/065 of the Ethical Committee for Laboratory Animal Care Charles Darwin France).

### Histopathological studies

Tissues were formalin fixed, embedded in paraffin and sectioned for histological analysis with hematoxylin–eosin staining. Mice were categorized into three classes: (i) with patent diffuse lymphoma, (ii) with microproliferation of lymphoma cells limited to the spleen, and (iii) with no histological evidence of lymphoma.

### Immunohistochemical studies

Immunohistochemical staining for Mlh1, Msh2, Msh6 and Pms2 proteins was performed in lymphoma cells from the single *Msh2*^wt^ mouse that displayed an MSI lymphoma following Aza treatment. Briefly, 4 μm sections were incubated with monoclonal antibodies against MLH1 (1/20 dilution, clone G168–728, Pharmingen, San Diego, CA), MSH2 (1/25 dilution, clone FE11, Calbiochem, Oncogene Research Products, Cambridge, MA), MSH6 (1/25 dilution, clone 44, Becton Dickinson, Lexington, NC) and PMS2 (1/20 dilution, clone A16–4, BD PharMingen, Le Pont de Claix, France).

### Loss of heterozygosity (LOH) analysis

Normal (muscle) and tumor DNA were extracted for *Msh2* genotype analysis (DNA extraction kit Qiagen, Courtaboeuf, France). PCR products were resolved on agarose gels to assess loss of heterozygosity of the *Msh2* allele [9].

### Microdissection of mouse tumor tissue

The tumor cell component from four Aza50-treated *Msh2^wt^* mice was collected from 10 hematoxylin-eosin-stained 7-μm tissue sections with a laser-capture microdissection system (PALM Laser, Zeiss, LePecq, France).

### Msh2 gene sequencing

Total RNA from the single Aza-treated *Msh2*^wt^ that displayed an MSI lymphoma tissue sample (two tumor sites and the corresponding normal tissue) was analyzed. Reverse transcription–PCR and sequencing were performed as previously reported [9], except that electrophoresis was performed for each amplified fragment and this was extracted specifically with a Gel Extraction Kit (Qiagen, Redwood City, USA). A second amplification was performed on these fragments according to the same PCR protocol in order to magnify the sequence signal and reduce noise.

### MSI testing

Polymerase chain reaction analysis of three noncoding mononucleotide repeats (A22, A24, T40) was used to determine the MSI status of tumor tissues [9]. Amplified PCR products were separated by capillary electrophoresis on an ABI PRISM 3100 Genetic Analyzer and analyzed using GeneMapper software (version 3.7, Aplera). Tumors were scored as MSI if the length of at least one mononucleotide repeat marker in tumor DNA differed from that observed in normal tissue DNA obtained from the same animal.

### Kaplan-Meier survival curves and statistical analysis

Time of death was recorded for each mouse that was found dead or had been sacrificed. Kaplan-Meier survival curves were generated using Graphpad Prism software (version 5, San Diego, CA). All statistical tests were two-sided.

### Characterization of lymphoproliferative disorders (LPD) in patients with inflammatory bowel disease (IBD)

A total of 19,486 patients were enrolled in the CESAME cohort, a French nationwide prospective observational cohort spanning May 2004 to June 2005 to assess the risk of LPD in patients with IBD treated with thiopurines [14]. MSI status was evaluated in 13 cases of LPD from paraffin material using a fluorescent multiplex system comprising 5 quasimonomorphic mononucleotide repeats (BAT-25, BAT-26, NR-21, NR-24 and NR-27) [21] and expression of MMR proteins was evaluated by immunohistochemistry [4]. LPD were classified according to the WHO classification 2001 and tested for Epstein Barr virus by immunohistochemistry or *in situ* hybridization.

## SUPPLEMENTARY FIGURES AND TABLES


