# Supplementary material for: Azathioprine induction of tumors with microsatellite instability: Risk evaluation using a mouse model
Source: Oncotarget. 2015 Aug 17;6(28):24969–77. doi: 10.18632/oncotarget.4638 (PMC4694807; doi:10.18632/oncotarget.4638)
Supplement: Supplementary file 1 [file oncotarget-06-24969-s001.pdf]

SUPPLEMENTARY FIGURES AND TABLES

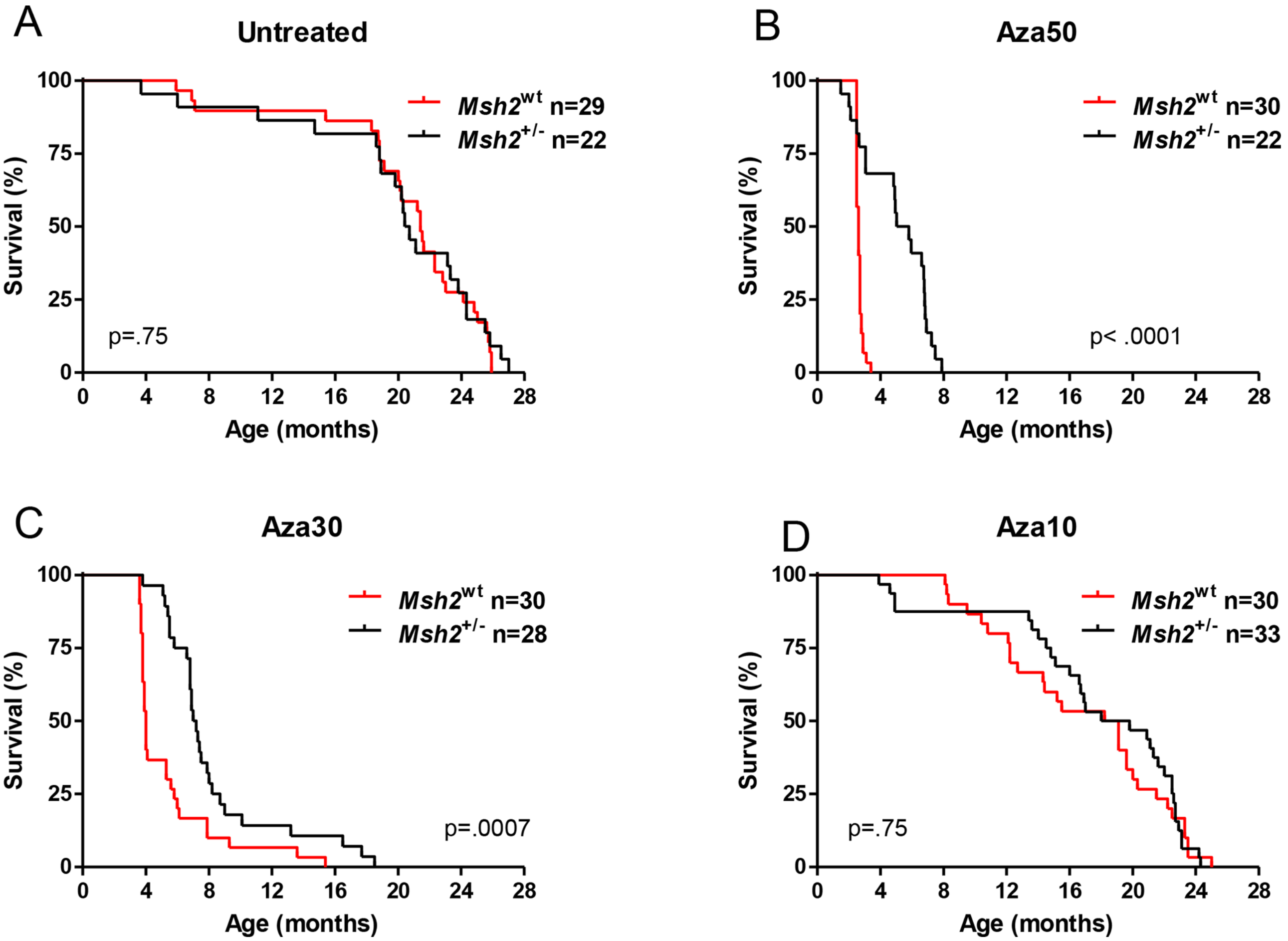

Supplementary Figure S1: Effect of *Msh2*$^{+/-}$ and *Msh2*^{wt} genotypes on mouse survival (Kaplan-Meier analysis) according to treatment. As expected, no difference in survival was observed between untreated *Msh2*$^{+/-}$ and *Msh2*^{wt} mice **A.** consistent with the normal lifespan previously reported for *Msh2*$^{+/-}$ mice. After Aza50 **B.** or Aza30 **C.** treatment, *Msh2*^{wt} mice showed significantly shorter survival than *Msh2*$^{+/-}$ mice. This was not seen at the lowest Aza dosage **D.** however, suggesting that Aza10 treatment allowed escape from toxicity in *Msh2*^{wt} mice. Aza10=azathioprine 10mg/L; Aza30=azathioprine 30mg/L; Aza50=azathioprine 50mg/L. All *P* values are two-sided. Log-rank test.

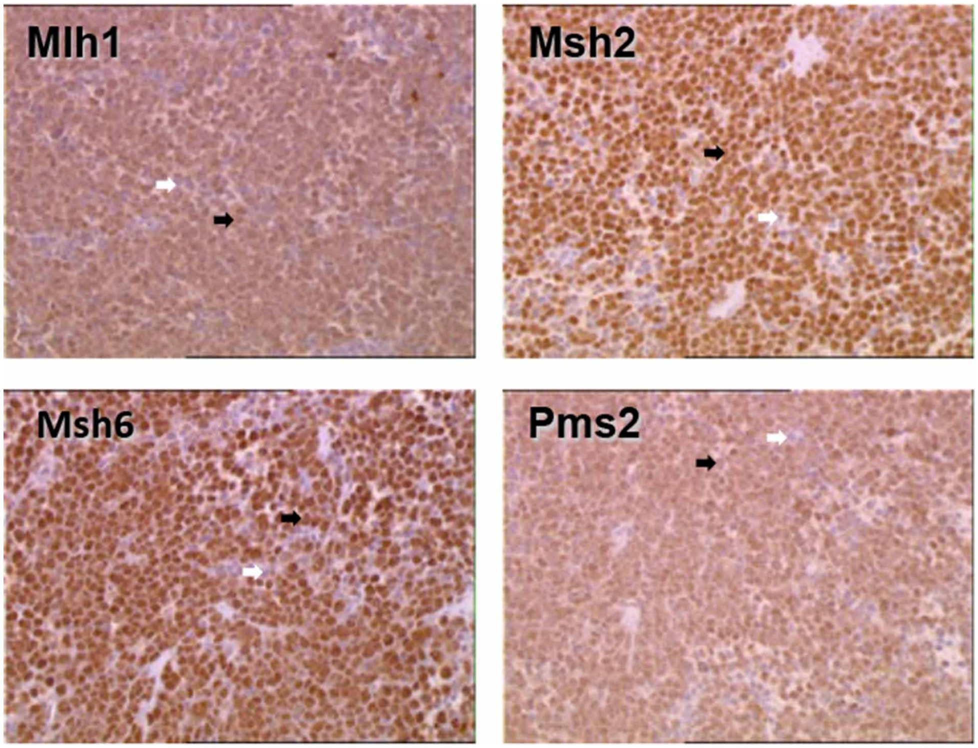

Supplementary Figure S2: Immunohistochemistry of spleen tissue sections from a non-MSI / MMR-proficient murine lymphoma arising in a Aza10-treated *Msh2*^{wt} mouse (mouse C1). Tumor cells (black arrows) stained positively for the four MMR proteins. Normal lymphocytes (white arrows) showed less pronounced staining of MMR proteins due to their lower proliferative rate. Antibodies against Mlh1 and Pms2 resulted in a less intense staining of tumor cells.

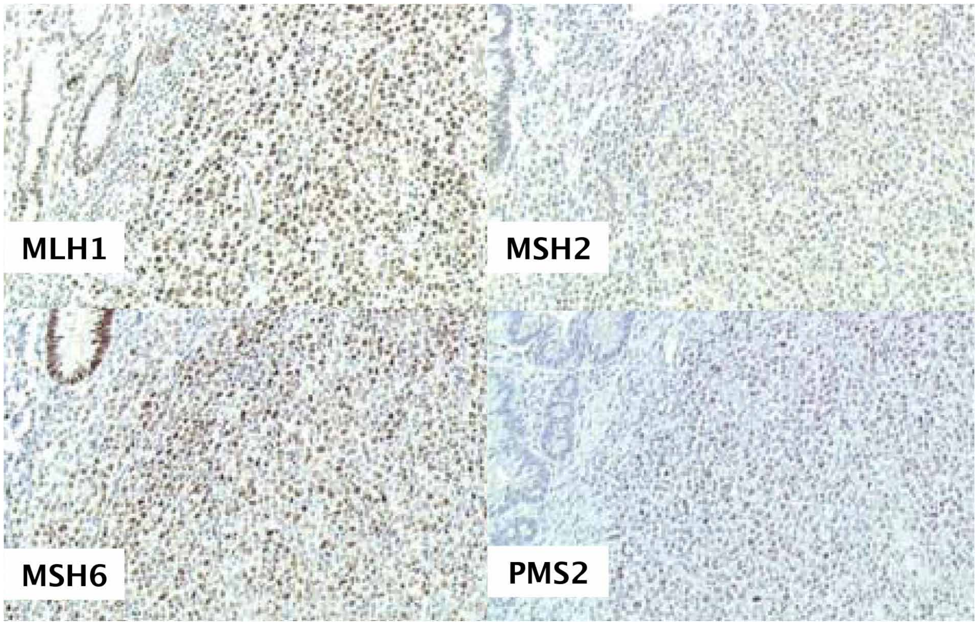

Supplementary Figure S3: Immunohistochemical analysis of MMR protein expression in tumor cells from a patient with inflammatory bowel disease that developed an iatrogenic lymphoproliferative disorder with an MSI phenotype. Tumor cells stained positively for the four MMR proteins. This tumor was not EBV-driven and occurred in a patient treated by imurel (see Supplemental Table S2).

Supplementary Table S1: Microsatellite analysis in tumor samples from *Msh2^wt^*, *Msh2^ko^* and *Msh2^+/–^* mice

Supplementary Table S2: Clinical and biological data associated with lymphoproliferative disorders in inflammatory bowel disease

Sex	IBD	Age	Extra nodal localization	Type	EBV status	Expression of the 4 MMR proteins	Microsatellite status	Imurel-treatment
M	CD	68	–	FL	ND	+	non-MSI	–
M	UC	62	–	FL	–	+	non-MSI	–
F	IC	76	Skin	DLBCL	–	+	NE	–
M	CD	56	–	FL	+	+	NE	+
M	CD	54	GI	FL	–	+	non-MSI	–
F	UC	75	–	DLBCL	ND	+	NE	–
F	CD	37	GI	DLBCL	+	+	non-MSI	+
F	CD	54	–	p-LPD B	+	+	non-MSI	+
M	CD	20	GI	DLBCL	+	+	NE	+
M	CD	56	GI	DLBCL	–	+	MSI	+
F	CD	79	GI	DLBCL	–	+	non-MSI	+
M	CD	52	Liver	HD-like LPD B	+	+	non-MSI	+
M	CD	45	Bone marrow	NHL B (MCL?)	ND	+	NE	+

IBD, inflammatory bowel disease; CD, Crohn disease; UC, ulcerative colitis; IC, indeterminate colitis; +, present; –, absent; ND, not done; NE; not evaluable; GI, gastrointestinal; DLBCL, diffuse large B-cell lymphoma; FL, follicular lymphoma; p-LPD B, polymorphous lymphoproliferative disorder B; MCL, Mantle cell lymphoma; NHL B, non-Hodgkin B-cell lymphoma; HD-like LPD B, Hodgkin disease-like lymphoproliferative disorder B; EBV, Epstein Barr virus; MSI, microsatellite instable.
